# Thermomechanical Behavior of Polymer Composites Based on Edge-Selectively Functionalized Graphene Nanosheets

**DOI:** 10.3390/polym10010029

**Published:** 2017-12-26

**Authors:** Ki-Ho Nam, Jaehyun Cho, Hyeonuk Yeo

**Affiliations:** 1 Institute of Advanced Composite Materials, Korea Institute of Science and Technology (KIST), Jeonbuk 565-902, Korea; khnam@kist.re.kr (K.-H.N.); jaehyun0119@kist.re.kr (J.C.); 2 Department of Chemistry Education, Chemistry Building, Kyungpook National University, 80, Daehak-ro, Buk-gu, Daegu 41566, Korea

**Keywords:** graphene, polyimide, polymer composite, thermo-mechanical properties

## Abstract

In this study, we demonstrate an effective approach based on a simple processing method to improve the thermomechanical properties of graphene polymer composites (GPCs). Edge-selectively functionalized graphene (EFG) was successfully obtained through simple ball milling of natural graphite in the presence of dry ice, which acted as the source of carboxyl functional groups that were attached to the peripheral basal plane of graphene. The resultant EFG is highly dispersible in various organic solvents and contributes to improving their physical properties because of its unique characteristics. Pyromellitic dianhydride (PMDA) and 4,4′-oxydianiline (ODA) were used as monomers for constructing the polyimide (PI) backbone, after which PI/EFG composites were prepared by in situ polymerization. A stepwise thermal imidization method was used to prepare the PI films for comparison purposes. The PI/EFG composite films were found to exhibit reinforced thermal and thermo-mechanical properties compared to neat PI owing to the interaction between the EFG and PI matrix.

## 1. Introduction

In recent years, polymer composites using graphene derivatives as filler have been studied with the aim of practical application in a wide range of academic and industrial fields [1,2,3,4,5]. In addition to their superior mechanical and electrical properties, because of the advantage of easily granting suitable physical and chemical characteristics for specific purposes, utilizing them as materials with excellent future prospects has been attracting much attention [6,7,8,9,10,11,12,13,14,15]. However, graphene as a filler component has a tendency to readily aggregate, and this becomes a major disadvantage in that it is difficult to conjugate its inherently excellent properties. Therefore, dispersing these derivatives uniformly in a polymer matrix rather than maintaining their superior properties has become a priority [16,17,18,19,20]. Accordingly, various methods have been investigated with the aim of achieving a high degree of dispersibility even if this is at the cost of reduced properties [21,22,23,24,25,26]. One of the most convincing ways is the chemical functionalization of the graphene, which makes it possible to improve the dispersibility to allow production on an industrial scale. However, since the method produces products with a variety of defects, extensive deterioration of the physical properties occurs. This introduces many problems in terms of their utilization as filler such as that they either have a very small reinforcement effect or no effect. In this regard, polymer composites using graphene fillers modified by the traditional chemical methods are problematic in that they are actually unable to provide any reinforcement. As a result, the key to a successful strategy for developing graphene polymer composites (GPCs) is the enhancement of their dispersibility and the suppression of defects. These two characteristics are almost inversely related.

With this in mind, many researchers have focused on finding a novel method to physically disperse graphene sheets that is sufficiently scalable to enable the mass production of GPCs [27,28,29,30,31]. In particular, one of the promising methods is that of Jeon et al. who reported that chemical functional groups could be effectively introduced at the edge of natural graphite to yield modified graphene through a physical process using ball milling with dry ice [32]. This method holds promise as a very good alternative for a chemical solution process to improve the dispersibility while retaining the unique excellent properties of graphene. This is because the method selectively introduces functional groups at the edge of graphene rather than in the basal plane of graphene. However, the application of edge-selectively functionalized graphene (EFG) to GPCs remains relatively uncommon.

In this paper, we report a series of GPCs using EFG as a reinforcement filler and polyimide (PI), which is one of the outstanding polymer materials, as a matrix polymer [33,34,35]. Especially, as the EFG filler has a high terminal carboxylic acid ratio, our GPCs would exhibit a strong direct and/or indirect interaction with the amine moieties of polyimides during in situ polymerization [18,19,36]. This effect is expected to facilitate homogeneous mixing as a result of the interactions between the EFG and PI chains, and also to improve the cohesion between the polymer chains, thereby affecting the thermodynamic properties. To grant the interactions to GPCs, there have been many previous studies such as introducing amines moiety in graphene [18,19]. However, the methods created a lot of defects in graphene for the introduction of functional groups, and the chemical reactions at several stages were essential. However, EFG is prepared via a very simple one-step reaction that allows the introduction of specific functional groups from natural graphite with low defects, which is practically usable. In addition, we controlled the level of defects on the filler by modifying the previous report. As a result, we succeeded in obtaining GPCs with greatly enhanced thermal and thermo-mechanical properties. Furthermore, the use of EFG in applications is expected to provide a new perspective for manufacturing GPCs.

## 2. Materials and Methods 

### 2.1. Materials

Graphite was acquired from Alfa Aesar (Graphite powder, natural, briquetting grade, −100 mesh, 99.9995%) and used without any purification. Dry ice was purchased from Taekyung Chemical Co., Ltd., Seoul, Korea. In addition, pyromellitic dianhydride (PMDA, >98%) and 4,4′-oxydianiline (ODA, >97%) were acquired from Tokyo Chemical Industry Co., Ltd., Tokyo, Japan and used as received. *N*-Methyl-2-pyrrolidone (NMP) was purified by a two-column solid-state purification system (Glass-contour System, Joerg Meyer, Irvine, CA, USA).

### 2.2. Experimental Procedure

#### 2.2.1. Preparation of EFG

Edge-selectively functionalized graphene (EFG) was synthesized based on a modification of the procedure reported by Baek et al. [32]. Before the ball-mill process involving pristine graphite, the graphite was vacuum dried at 80 °C for 24 h. Then, in a closed and perfectively sealed stainless steel jar, 5 g of graphite was simply milled with 50 g of dry ice, which is only half the amount recommended in the previous report. Milling was achieved by using a planetary mono ball-mill machine (Pulverisette 6, Fritsch, Germany) at 480 rpm with 50 g of stainless steel balls 5 mm in diameter for 48 h. Because the internal pressure increased to about 100 bar, the carbon dioxide is released from the dry ice in the form of a supercritical fluid; therefore, a smaller amount of dry ice was sufficient. The resulting-EFG was further purified by Soxhlet extraction with an aqueous solution of 1 M HCl to completely acidify the carboxyl derivatives. Then, the products were washed with distilled water and freeze-dried at −120 °C for 72 h.

#### 2.2.2. Preparation of PI/EFG Composites

The PI/EFG composites were fabricated via in situ polymerization and subsequent thermal cyclic dehydration. EFG powder (filler loadings of 0.1–3 wt %) was dissolved in NMP using an ultrasonic bath for 30 min. Next, equivalent molar ratios of ODA (2 mmol) and PMDA (2 mmol) were dissolved in the EFG dispersion with continuous stirring for 24 h in an argon atmosphere. The poly-condensation was identical to that of the viscous polyamic acid (PAA)/EFG solution. After degassing with a vacuum pump, the resulting mixture was spin-coated at 500 rpm onto a fused silica substrate and then pre-baked at 90 °C/2 h and 150 °C/1 h in vacuo. The PAA/EFG films were thermally imidized to PI/EFG composites under specific thermal curing at 200 °C/1 h, 250 °C/30 min, and 300 °C/30 min in a furnace under an argon atmosphere.

### 2.3. Measurements

The morphologies of the samples were investigated by scanning electron microscopy (SEM, Nova NanoSEM, FEI, Hillsboro, OR, USA) analysis. Fourier transform-infrared (FT-IR) spectra were obtained by using KBr pellets (FT-IR spectrometer, Vertex80v, Bruker, Billerica, MA, USA) in the range 400 to 4000 cm^−1^. Raman spectroscopy was carried out using a micro-Raman spectrometer (inVia Raman spectrometer, Renishaw, Wotton-under-Edge, UK) with a laser having a 514.5 nm light source with an output of 0.15 mW. Thermogravimetric analysis (TGA) was performed on a Q50 machine (TGA, TA Instruments, New Castle, DE, USA) with an N_2_ gas flow at a heating rate of 10 °C/min. Dynamic mechanical analysis (DMA) was conducted by using PI and GPC films (30 mm length, 5 mm wide, and ca. 30 μm thickness) on a Q800 machine (DMA, TA Instruments, New Castle, DE, USA) at a heating rate of 3 °C/min with a load frequency of 1 Hz in air. Electrical conductivity was measured by SM-8311 machine (HIOKI, Nagano, Japan) under 500 V.

## 3. Results and Discussion

### 3.1. Synthesis and Characterization of Edge-Selectively Functionalized Graphene (EFG)

#### 3.1.1. Synthesis of EFG

The filler, edge-selectively functionalized graphene (EFG), was prepared by using a slightly modified version of the reported ball-milling process [32]. Similar to the published method, the EFG was functionalized by ball-milling and additional refinement; however, the amount of dry ice was halved to reduce the degree of functionalization as intended. Additionally, the processing time was controlled to prepare larger particles. In particular, ball-milling functionalization using dry ice is known to produce high carboxylic acid derivative content. This derivative can either interact directly with the amine moieties of the co-monomer component by way of covalent bonding during in situ polymerization, or interact indirectly with the imide groups of the resulting PIs by way of hydrogen bonding [18,19,36].

**Figure 1:**
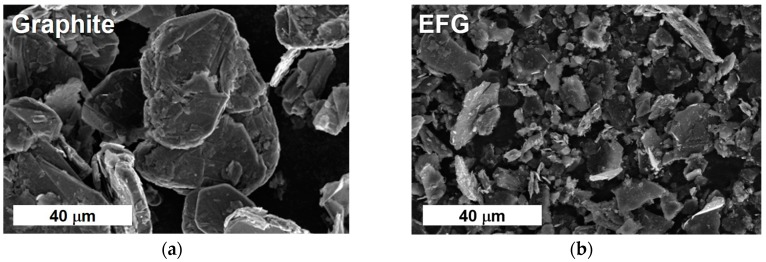
Scanning electron microscopy (SEM) images: (**a**) Natural Graphite; (**b**) Synthesized edge-selectively functionalized graphene (EFG).

The morphological features of the synthesized EFG and those of pristine natural graphite were compared by SEM observation (Figure 1). The platelets of pristine graphite have an irregular shape of known size, which is the typical natural form. The sheets of synthesized EFG had a similar irregular shape, but the size was much smaller and the surface smoothed. However, as we intended, the size was controlled to be of the order of micrometers even though we first reported the sample to be prepared in ~500 nm size because our experiment was conducted under more mild conditions.

#### 3.1.2. Characterization of EFG

**Figure 2:**
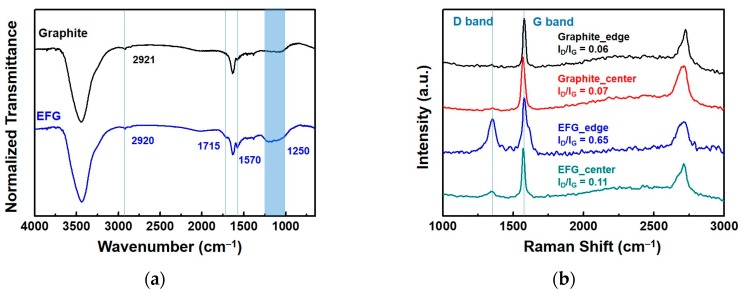
Characterization of EFG and graphite: (**a**) Fourier transform-infrared (FT-IR) spectra; (**b**) Raman spectra and *I*_D_/*I*_G_ ratio.

The chemical composition of EFG was analyzed by investigating the functionalization results by FT-IR and Raman spectroscopy (Figure 2). The FT-IR spectra (Figure 2a) revealed peaks for the EFG that were not observed in pristine graphite. Specifically, we could identify a peak at 1715 cm^−1^ assigned to C=O stretching and a broad peak around 1250 cm^−1^, which indicated the coexistence of C–OH (hydroxyl), C–O–C (epoxy), and O=C–OH (carboxyl), respectively [32]. In addition, the peaks at 2920 and 1570 cm^−1^ observed in both EFG and graphite were assigned to *sp*^2^ C–H and aromatic C–C stretching, respectively. These results indicated that the chemo-physical method for functionalizing graphene using a ball-mill and dry ice could efficiently introduce functional groups, especially carboxyl groups, without the use of strong acids such as sulfuric acid or nitric acid and explosive oxidants.

In addition, EFG was further characterized by micro-Raman spectroscopy. The measurements were carried out at both the edge and the basal plane of the pristine graphite and EFG. In the case of the graphite, the *I*_D_/*I*_G_ ratio (i.e., the ratio of the intensity of the D-band at 1350 cm^−1^ to that of the G-band at 1585 cm^−1^) was almost the same for both the edge and the plane. However, interestingly, EFG exhibited a significant difference in the values of the *I*_D_/*I*_G_ ratio. The ratio at the edge of EFG was 0.65, which is a significant increase, whereas the value at the center of the sheet plane was almost maintained compared to the ratio for pristine graphite. These results suggest that the composition of the edge was selectively changed into more disordered structures with the *sp*^2^-defects mainly derived from carboxylation [18].

### 3.2. Fabrication and Thermo-Mechanical Properties of PI/EFG Composites

#### 3.2.1. Fabrication of PI/EFG Composites

**Figure 3:**
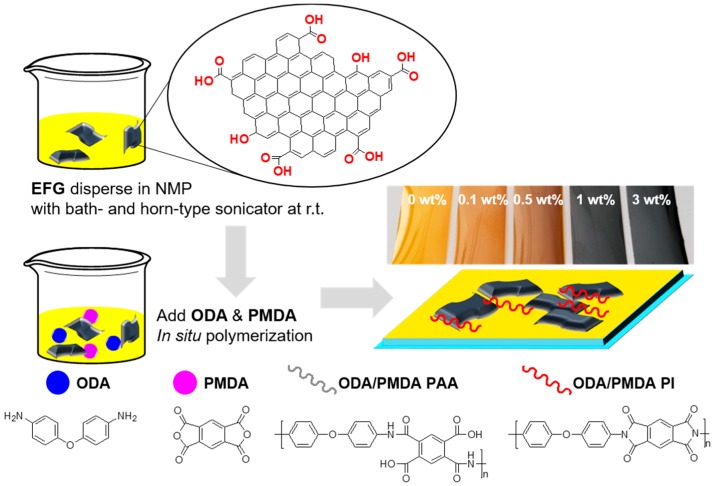
Fabrication process of PI/EFG composites.

The application of EFG was studied by fabricating a series of polymer composites consisting of a typical aromatic polyimide as a matrix polymer and EFG as filler. The detailed procedure is described in Figure 3. First, the EFG was dispersed in NMP with sonication. Subsequently, the poly(amic acid) solution as a pre-polymer for GPC fabrication with various EFG content was prepared by direct in situ polymerization with pyromellitic dianhydride (PMDA) and 4,4′-oxydianiline (ODA), which are the most common monomers for PI synthesis. Lastly, after the GPC films of the PAA states were fabricated, gradual thermal treatment resulted in cured polyimide films. The GPCs were fabricated in four varieties with EFG filler content of 0.1, 0.5, 1.0, and 3.0 wt %, respectively. In addition, as a reference for comparison, the pure PI film was also prepared under the same conditions. All GPC and pure PI films were obtained in the form of a flexible and transparent film that resembled a typical PI film. As the EFG content increased, the GPC became slightly translucent.

#### 3.2.2. Characterization of PI/EFG Composites

**Figure 4:**
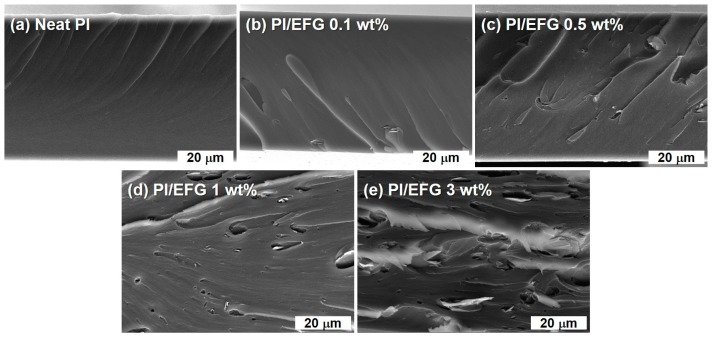
SEM images of GPCs: (**a**) Neat PI; (**b**) PI/EFG 0.1 wt %; (**c**) PI/EFG 0.5 wt %; (**d**) PI/EFG 1 wt %; (**e**) PI/EFG 3.0 wt %.

**Figure 5:**
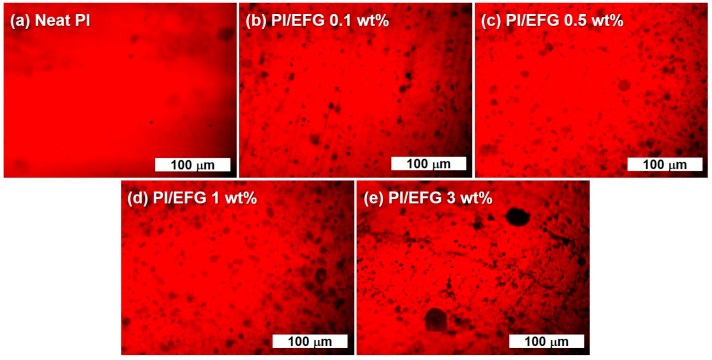
Optical microscope (OM) images of GPCs: (**a**) Neat PI; (**b**) PI/EFG 0.1 wt %; (**c**) PI/EFG 0.5 wt %; (**d**) PI/EFG 1 wt %; (**e**) PI/EFG 3.0 wt %.

The cross-sectional images of the GPC films on the fractured surface were observed by scanning electron microscopy, confirming the morphology of the films and the distribution of the EFG filler (Figure 4). The surface of pure PI appeared smooth without any roughness, but the GPCs had a more coarse morphology. The GPCs with filler loadings of 0.1, 0.5, and 1 wt % exhibited a surface roughness that was almost the same as that of pure PI, although the surface tended to become slightly coarse as the amount of EFG increased, indicating that the EFGs were well dispersed in the matrix polymer. However, the GPC with 3 wt % EFG had an apparently harsh surface morphology in accordance with its high filler loading. In addition, the aggregation of filler platelets was also observed. Even though a little aggregation was observed at a high filler loading, considering that the EFG was not highly functionalized, the GPCs were successfully fabricated with well-dispersed EFGs originating from their direct and/or indirect interfacial interaction. The same trends were also conformed in the optical microscopy images measured to detect the dispersion quality of the fillers in a larger scale (Figure 5). In the 1 and 3 wt % samples, it was observed that the fillers were partially aggregated, but overall, the uniformly dispersed states were confirmed.

#### 3.2.3. Thermal and Thermo-Mechanical Properties of PI/EFG Composites

**Figure 6:**
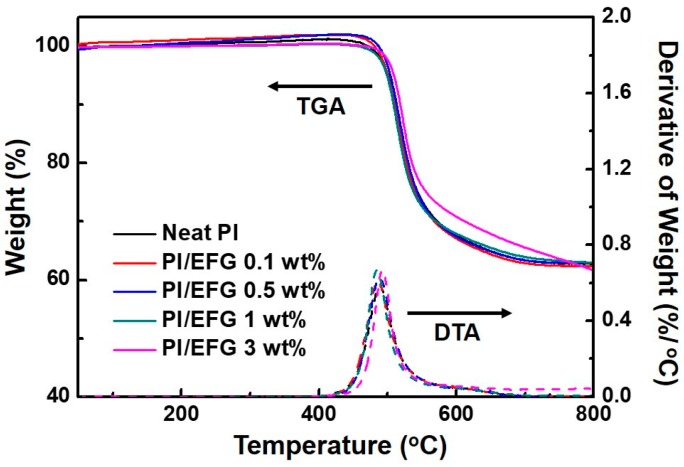
Thermogravimetric analysis (TGA) (solid lines) and derivative weight loss curves (dashed lines) of neat PI and PI/EFG composites.

The thermal and thermo-mechanical properties of the GPCs were examined by thermogravimetric analysis (TGA) and dynamic mechanical analysis (DMA) (Table 1). The thermal stability curves of the GPCs evaluated by TGA appeared almost similar regardless of the filler content because similar thermograms were observed (Figure 6). In fact, the filler loadings of the GPCs were so low that they naturally exhibited similar thermal decomposition behavior. However, upon examining the detailed curves, they tended to display increased decomposition temperatures, i.e., *T*_d,5%_ and *T*_d,10%_, according to the filler loadings. These results could be interpreted in two ways: EFG is thermally very stable, so the decomposition temperature of the GPCs increases according to their filler content, or, the enhancement is derived from the interaction between EFG and PI chains. As far as the char yields after thermal decomposition up to 800 °C are concerned, the latter effect is considered to be large. Therefore, as we intended, the increased thermal stability could be attributed to the direct/indirect interaction between EFG and PI chains as a consequence of covalent and hydrogen bonding with the carboxylic acid group on the edge of EFG [18,19,36].

**Figure 7:**
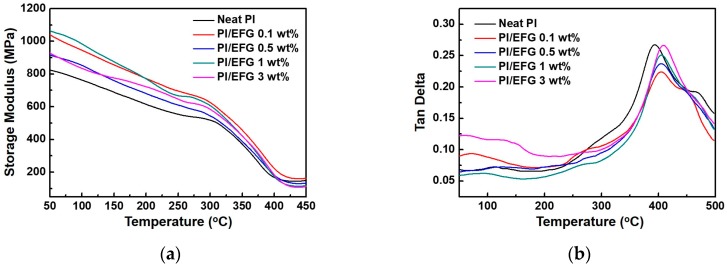
Dynamic mechanical analysis (DMA) curves of neat PI and PI/EFG composites: (**a**) Storage modulus; (**b**) tan *δ*.

The DMA measurements of the GPCs were carried out in order to confirm the interaction between the functional groups on EFG and the PI polymer chains as well as to investigate their thermo-mechanical properties (Figure 7). First, the GPCs had a larger storage modulus than pure PI due to the reinforcing effect of the EFG. In addition, the tendency of the modulus to increase was reversed and a decrease was observed owing to the aggregation phenomenon that occurred in the GPC containing 3 wt % EFG. Interestingly, there was a difference in the glass transition temperature (*T*_g_) of the GPCs calculated by the DMA measurements. In general, polymer composites containing heterogeneous fillers have lower *T*_g_ values than those of pure polymers due to the effect of the fillers on the inter-chains that causes the interaction between the polymer chains to weaken. In contrast, the GPCs with EFG showed an increasing tendency for *T*_g_ as the filler content increased. In particular, the *T*_g_ value increased by more than 10 °C for all the GPCs. This significant enhancement in *T*_g_ is only possible when specific forces, such as hydrogen bonding or additional chemical bonding, are in effect between the polymer chains [37]. These reinforcement effects would occur additively as a result of the specific interaction between the functional groups at the edge of EFG and the imide groups of the PI chains, and the additional effect of the EFG itself forming a covalent bond with the amine moieties to act as the center in the network branches during in situ polymerization.

**Figure 8:**
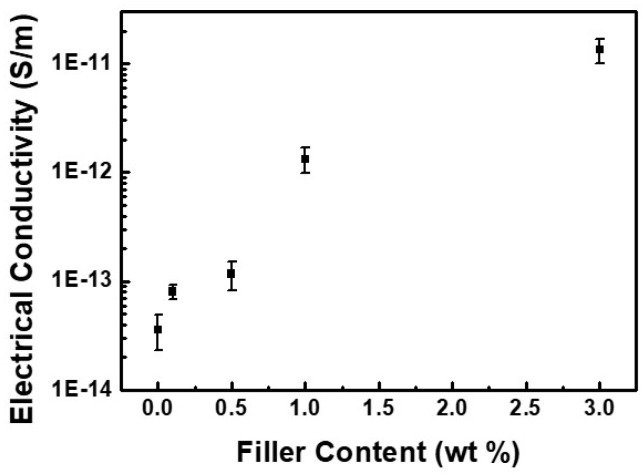
Electrical conductivity of neat PI and PI/EFG composites.

In addition, in order to investigate further strengthening effect by EFG, the electrical conductivity was measured (Figure 8). Since it is well known that graphene shows exceptionally high electron mobility and electrical conductivity, the composites containing EFG would be also expected to exhibit high electrical conductivity. As expected, the electrical conductivity of the GPCs increased significantly as the EFG content increased. Considering that the amount of filler introduced is up to 3 wt %, which is insufficient to form a percolated pathway for electrical conducting, the conductivity increased by about 10^3^ orders of magnitude to neat polyimide suggested that introduction of EFG would effectively build partial conducting chain. This reinforced result should have be attributed to both of the interaction between PI chains and EFGs and evenly distributed EFGs in GPCs.

## 4. Conclusions

In this work, graphene with carboxyl functionalities at the edges of graphene platelets was prepared by the modified ball-milling method in the presence of dry ice. Our method successfully produced a less-functionalized EFG with a larger grain size as confirmed by location-selective micro-Raman spectroscopy.

We utilized the resultant EFG as filler to fabricate GPCs for potential application in such as electronic, automotive, airline, space, defense, and construction industries. A series of GPCs was prepared by varying the EFG contents and using aromatic PI as a matrix polymer, which is one of the representative polymers capable of interacting effectively with the filler. The GPCs were prepared via traditional two-step thermal imidization between PMDA and ODA. During the process, the poly(amic acid) compounds were obtained and used to fabricate the GPC films. The resulting GPC films showed a clear surface morphology except for the 3 wt % sample, which meant that the EFG platelets were well dispersed. In particular, the fact that the EFGs were well dispersed in the GPCs led to the following three notable enhancements compared with pure PI. First, in terms of their thermal properties, the GPCs exhibited improved thermal stabilities. Furthermore, their mechanical properties observed by DMA were enhanced. Lastly, the *T*_g_ values of the GPCs significantly increased. These reinforcements could be attributed to the additional covalent bonds between the EFGs and amine moieties of the PI and the electrostatic interaction between the EFGs and PI chains, as initially designed.

## Figures and Tables

**Table 1:** Thermo-mechanical properties of PI/EFG composites.

Sample (wt %)	*T*_d,5%_ Determined by TGA [°C] ^1^	*T*_d,10%_ Determined by TGA [°C] ^2^	Char Yield [%] ^3^	*T*_g_ Determined by DMA [°C] ^4^
Neat PI	500.0	509.7	62.0	393.5
PI/EFG 0.1 wt %	499.9	509.0	62.9	404.6
PI/EFG 0.5 wt %	502.2	512.6	63.3	405.4
PI/EFG 1.0 wt %	505.4	514.3	63.8	405.9
PI/EFG 3.0 wt %	509.6	517.6	61.8	408.6

^1^ The decomposition temperatures for 5% weight loss from TGA. ^2^ The decomposition temperatures for 10% weight loss from TGA. ^3^ Weight percentage of residues at 800 °C. ^4^ The values were calculated by the peak points of the Tan *δ* graph.
